# PubChem3D: Diversity of shape

**DOI:** 10.1186/1758-2946-3-9

**Published:** 2011-03-21

**Authors:** Evan E Bolton, Sunghwan Kim, Stephen H Bryant

**Affiliations:** 1 National Center for Biotechnology Information, National Library of Medicine, National Institutes of Health, Department of Health and Human Services, 8600 Rockville Pike, Bethesda MD 20894, USA

## Abstract

**Background:**

The shape diversity of 16.4 million biologically relevant molecules from the PubChem Compound database and their 1.46 billion diverse conformers was explored as a function of molecular volume.

**Results:**

The diversity of shape space was investigated by determining the shape similarity threshold to achieve a maximum on the count of reference shapes per unit of conformer volume. The rate of growth in shape space, as represented by a decreasing shape similarity threshold, was found to be remarkably smooth as a function of volume. There was no apparent correlation between the count of conformers per unit volume and their diversity, meaning that a single reference shape can describe the shape space of many chemical structures. The ability of a volume to describe the shape space of lesser volumes was also examined. It was shown that a given volume was able to describe 40-70% of the shape diversity of lesser volumes, for the majority of the volume range considered in this study.

**Conclusion:**

The relative growth of shape diversity as a function of volume and shape similarity is surprisingly uniform. Given the distribution of chemicals in PubChem versus what is theoretically synthetically possible, the results from this analysis should be considered a conservative estimate to the true diversity of shape space.

## Background

Virtual screening of large chemical databases is now a routine practice in modern drug discovery [1-8]. One successful virtual screening approach is to compare the 3-D shape similarity of chemical structures using atom-centered Gaussian functions [9-11], *e.g*., as implemented in ROCS [12]. While this Gaussian-based approach to shape can perform hundreds or even thousands of chemical structure 3-D shape superposition computations per second per Central Processing Unit (CPU) core, even faster approaches with similar efficacy would be welcomed when searching a database of millions of chemical structures and (potentially) billions of conformers.

Attempts [13,14] have been made to use ROCS to identify reference shapes, which are then used to compute 3-D shape similarities at dramatically enhanced rates. One approach [13] created a binary "shape fingerprint" used much like traditional 2-D molecular connectivity fingerprints, where individual bits are "turned on" whenever the reference shape has sufficient shape similarity, as defined by the Shape Tanimoto (ST) in **Equation 1**, to the conformer being considered. Binary shape fingerprints, as an approach, were shown as a promising technique to encode the shape of a chemical structure conformer and achieve very fast 3-D similarity computation, but with the potential downside of not providing an actual 3-D conformer superposition and with no guarantee that the shape similarity values or result lists have any correlation with those provided by ROCS.(1)

where *V_AA _*and *V_BB _*are the self-overlap volume of molecules A and B and *V_AB _*is the overlap volume between them and the ST score ranges from 0 (for no shape similarity) to 1 (for identical shapes).

A second 3-D similarity approach using reference shapes [14] attempted to improve upon the first method by giving both a shape superposition and some assurance that the shape similarity ST is similar to that provided by ROCS. This was achieved by recognizing that two chemical structure conformers with similar 3-D shape align to a common reference shape in a similar fashion. By utilizing the 3 × 3 rotational matrix and XYZ translational vector that align a 3-D chemical structure conformer to a common reference shape (retained after shape fingerprint generation), one could generate a superposition between conformers for each common reference shape. Given that two similar conformers may have multiple common reference shapes, one may "replay" all the alignments to common reference shapes and pick one that yields the best shape superposition. This approach achieved a 100× fold performance improvement by avoiding any shape similarity computation when shapes were too dissimilar (*i.e*., there were no common reference shapes) and by avoiding any volume overlap maximization optimization computations. However, this methodology has its downsides. It only considered relatively small (<28 non-hydrogen atoms) and inflexible (<6 rotatable bonds) chemical structures and would not compute any shape similarity value when there was no common reference shape. Yet, in both studies [13,14], it was shown that the use of reference shapes may provide promise to dramatically improve the throughput of shape-based alignment methodologies.

The first work described above [13] considered data sets of "drug-like" molecules with 12-32 non-hydrogen atoms and conformer counts between 50,000 and 500,000 to examine the growth of shape space as a function of ST value. This growth was linear when considering the logarithm of the count of reference shape and chemical structures, whether using a single conformer or multiple conformers per structure. The second work [14] also considered reference shapes of "drug-like" molecules of similar size, using a much larger dataset of one million chemical structures and fifteen million conformers, but only at a single ST value, as opposed to a range of ST values. Still, both studies gave valuable insight into how shape space grows with "drug-like" molecules.

In this work, we seek to expand upon these earlier two efforts by exploring in more depth the rate of growth of shape space as a function of reference shape count, conformer volume, and ST value with a much larger data set of 16.4 million biologically relevant small molecules and their 1.46 billion diverse conformers. By improving upon the understanding of the relative growth of shape space of biologically relevant molecules, new or improved "shape fingerprint"-based methodologies might be developed.

## Results and Discussion

### 1. Conformer generation

**Figure 1:**
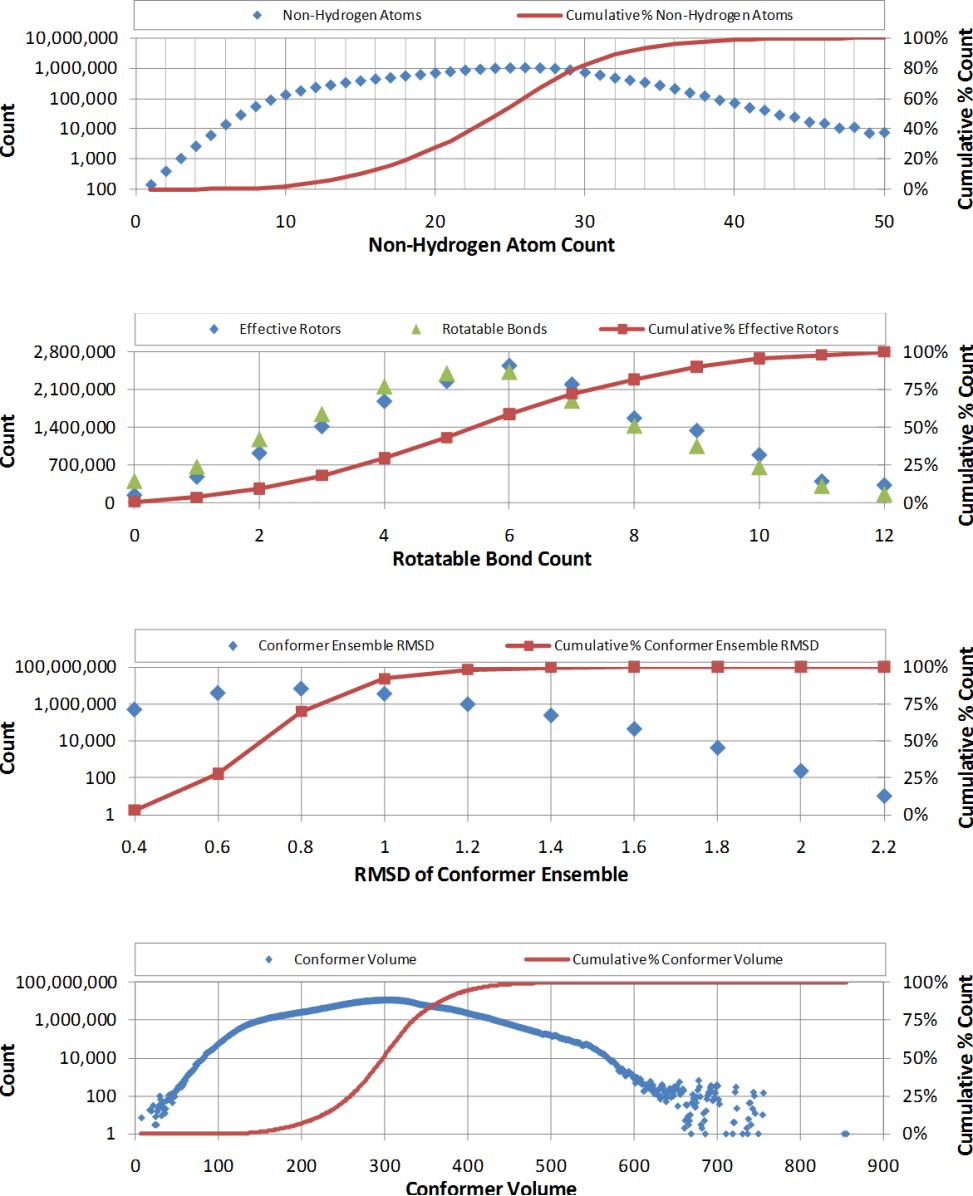
**The distribution of non-hydrogen atom count, rotatable bond count, conformer ensemble sampling RMSD, and conformer volumes (rounded to the nearest integer) of 1,465,813,269 conformers generated from 16,482,382 molecules in the PubChem Compound database**.

Conformers were generated for chemical structures in the PubChem Compound database[15-18] as described in the *Materials and Methods *section. This resulted in 16,482,382 3-D conformer ensemble models (as of February 2008) and 1,465,813,269 diverse conformers (an average of 89 conformers per compound). The distribution of the non-hydrogen atom count, rotatable bond count, sampling RMSD, and conformer volumes (rounded to nearest integers) for these are shown in Figure 1. The average count and standard deviation of non-hydrogen atoms was 24.5 +/- 6.8 with a mode of 26 (with 1,033,645 compounds). The average count and standard deviation of rotatable bonds was 5.5 +/- 2.6 with a mode of 6 (with 2,432,059 compounds). The average and standard deviation of the sampling RMSD for the conformer ensembles was 0.82 +/- 0.20 Å with a mode of 0.8 Å (for 6,939,072 conformer ensembles). The average and standard deviation of the conformer volume was 297 +/- 64 Å^3^. The most common volume among the conformers was 307 Å^3 ^(for 10,920,699 conformers) and 99% of the conformers have a volume between 130 and 487 Å^3^. In further analyses, we focused on the conformers whose volumes were between 75 and 575 Å^3^, corresponding to 99.99% of all conformers.

### 2. Generation of reference shapes per volume

The shape diversity of a particular conformer volume may be ascertained by clustering conformers of that volume with a certain shape diversity threshold (*ST^thresh^*), which controls the "minimum" distance between any two clusters, and then by counting the number of reference shapes, each of which represents a cluster centroid and all conformers within *ST^thresh ^*to the reference shape. [Note that the *ST^thresh ^*is the "maximum" ST value between clusters since the ST score is a *similarity *measure, not a *dissimilarity *measure.] If the clustering is performed using the same *ST^thresh ^*value for a volume range, the shape diversity as a function of each molecular volume size may be evaluated by the growth of the number of reference shapes. However, when a constant *ST^thresh ^*value is used across a range of volumes, each increase in the molecular volume may result in a very rapid growth of the shape space, and hence, the number of reference shapes per volume. This is not completely desirable as the computational cost of clustering effectively increases as the square (or worse) of the total count of reference shapes (especially when this count is large), when considering *N *reference shapes must be compared against *K *conformers and *N *<*K*, compelling one to keep the count of reference shapes to a manageable size for tractability purposes.

**Figure 2:**
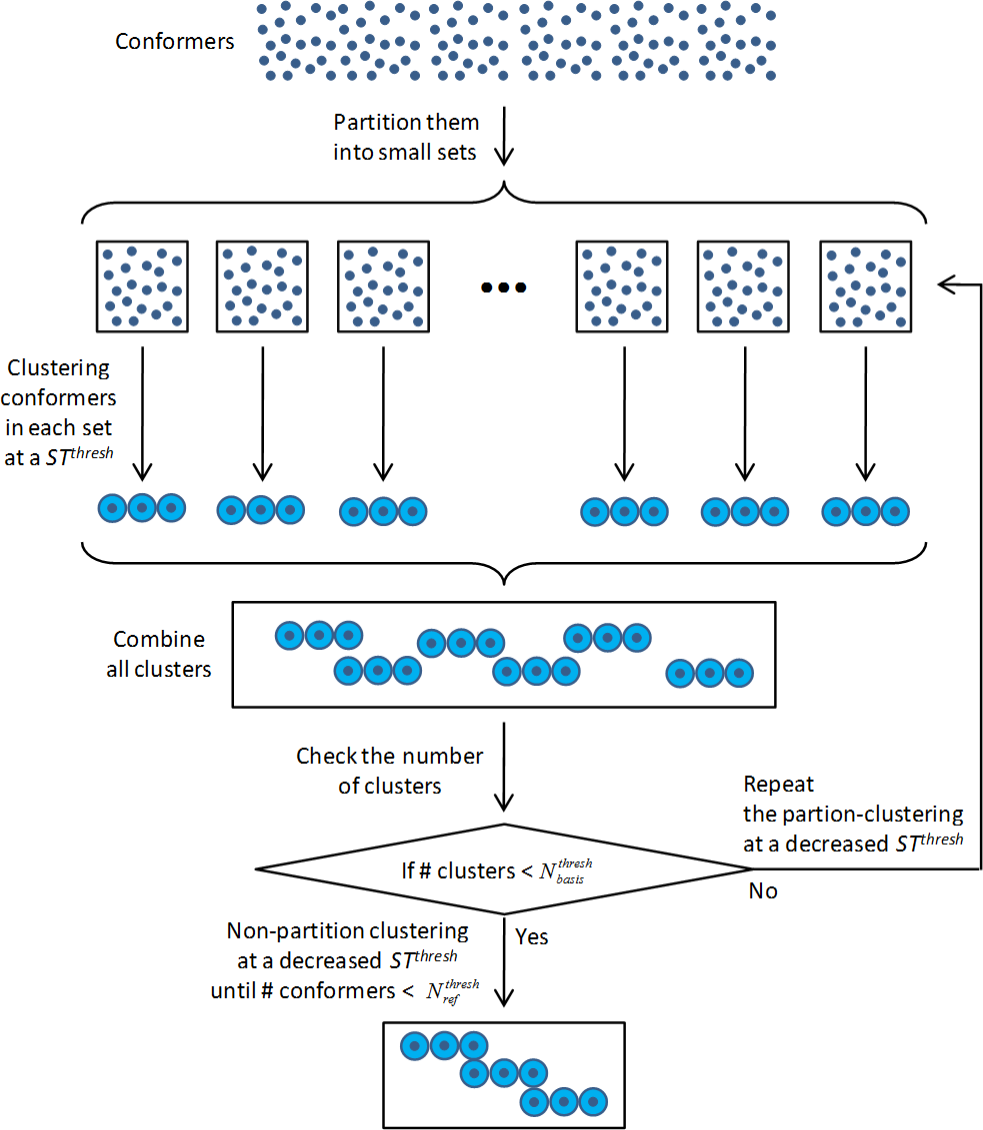
**Partition-clustering scheme used for generating the reference shapes for a given volume**.

To avoid excessive computational expense, we took an alternative approach (as described in Figure 2), in which the clustering for a given volume was performed with a dynamic *ST^thresh ^*value such that the resulting reference shape count became less than or equal to a certain number (chosen to be 200). In this manner, the number of reference shapes per volume was kept effectively constant (as an increase of ST by 0.01 would result in reference shape count above 200), while the growth of shape space as a function of volume is manifest by a decrease in *ST^thresh^*. The detailed procedure for clustering is explained in the *Material and Methods *section and the PubChem Compound ID of the resulting reference shapes can be found on the PubChem FTP site ftp://ftp.ncbi.nlm.nih.gov/pubchem/Compound_3D/ReferenceShapes/.

**Figure 3:**
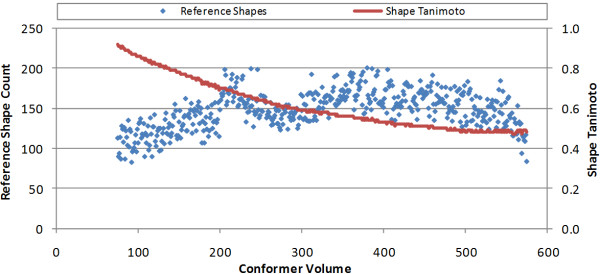
**The Shape Tanimoto value used as a shape diversity threshold (*ST^thresh^*) and the resulting reference shape counts as a function of volume**.

Figure 3 shows the *ST^thresh ^*value and the reference shape counts as a function of the conformer volume. The *ST^thresh ^*score decreases gradually and uniformly in the 75-575 Å^3 ^range from 0.92 (for V = 75 Å^3^) to 0.47 (for V = 558 Å^3^). In fact, this decrease is so smooth that one can predict the ST value in the volume range 75-575 Å^3 ^using only the conformer volume (**Equation 2**) with an R^2 ^value of 0.997.(2)

where *V *is the conformer volume and *ST^thresh ^*is the shape Tanimoto for the given volume to achieve 200 or fewer reference conformers. The slope of the *ST^thresh ^*curve shows that the increase in the cluster distances becomes slower as the conformer volume increases; however, this reduction may be an artifact of the input. The reason for this is relatively simple. This study only considered chemical structures found in PubChem and was restricted to 50 or less non-hydrogen atoms. Furthermore, the distribution of this non-hydrogen atom count had a maximum of 26. Conceivably, *ST^thresh ^*may decrease at a more rapid rate if the count of chemical structures in PubChem continued to increase as a function of non-hydrogen atom count across the entire range of non-hydrogen atom count, rather than hitting a maximum of 26. The net effect of this input artifact is that the *ST^thresh ^*curve in Figure 3 may be more linear than actually shown. We expect the entire curve as shown may shift and appear more linear as more theoretically possible and diverse chemical structures are considered; however, we believe the trends detailed in this work should still hold true, unless noted otherwise. Irrespective of the explanation provided, one should consider the curve shown in Figure 3 a conservative estimate of the absolute growth of shape space.

The reference shape count per volume was found to range from 83 (for V = 92 Å^3^) to the maximum allowed of 200 (for V = 380 Å^3^), and its average was 147.9. Interestingly, the *ST^thresh ^*curve does not reflect the maximum found in Figure 1 for conformer volume. In fact, the decrease in *ST^thresh ^*as a function of volume is very smooth, suggesting that the actual conformer count per volume, as shown in Figure 1, has little bearing on shape diversity, as shown in Figure 3. Or, put another way, the shape space of known chemicals is not near as diverse as chemical space, with a relatively small amount of reference shapes able to represent a large number of chemical structure conformers.

Another interesting observation is that a small change in *ST^thresh ^*has a large effect on reference count, as reflected in the somewhat periodic growth in shape references until the maximum value of 200 reference shapes is reached, cutting the reference shape count nearly in half. This can be roughly seen in the volume range 75-210 Å^3^and then again between 275-375 Å^3^. This reflects the use of 0.01 decrements in *ST^thresh ^*but also reflects anecdotal evidence seen when exploring the reference shapes, where each change in *ST^thresh ^*by 0.01 appeared to change the reference count by about a factor of two, much as observed by Haigh, *et al*. [13] This is only roughly seen in the reference shape counts as two things are changing, the volume and the *ST^thresh ^*value, and volume change involves a potentially variable change in shape space.

### 3. Generation of unique shapes for each volume

**Figure 4:**
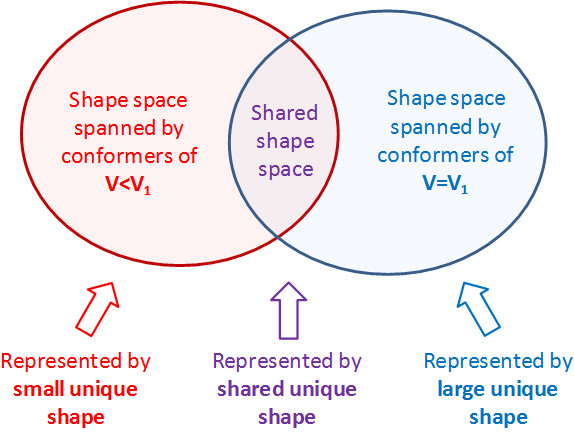
**The concept of unique shapes for V = V_1_, which cover the shape space spanned by the conformers whose volumes are less than or equal to V_1_**.

**Figure 5:**
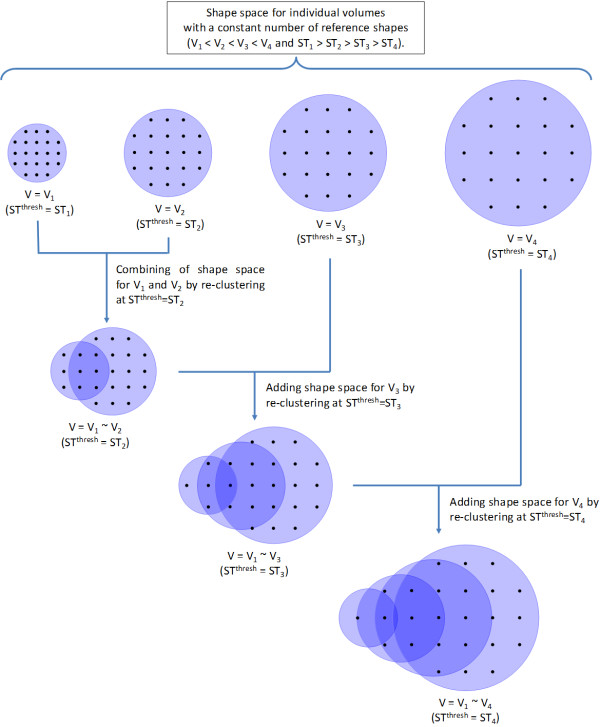
**Schematic illustration of the shape space expansion upon a conformer volume increase**. Blue circles represent the shape space spanned by conformers of a particular volume (V), and black dots represents reference shapes (for the individual shape spaces) or unique shapes (for combined shape spaces). *ST^thresh ^*indicates a Shape-Tanimoto (ST) value used as a shape diversity threshold.

Reference shapes generated for a given volume are guaranteed to not be closer than the corresponding *ST^thresh ^*value so that the ST similarity between any two reference shapes for that volume cannot be greater than *ST^thresh^*. However, it is still possible that two reference shapes of different volumes may be closer than *ST^thresh^*, implying that some portion of the shape space covered by reference shapes for V = V_1 _can also be shared by reference shapes for V ≠ V_1_. For this reason, we introduced the concept of the "unique shapes" for a given volume, defined as a non-overlapping set of conformers that cover the shape space spanned by the conformers whose volume is *smaller than or equal to *that volume (that is, V≤V_1_). As illustrated in Figure 4, the unique shapes were classified into three groups according to the shape space they cover: (1) the "large unique shapes", which cover the shape space spanned only by the conformers of V = V_1_, (2) the "small unique shapes", which cover the shape space spanned only by the conformers of V<V_1_, and (3) the "shared unique shapes", which cover the shape space spanned by the conformers of V = V_1 _*and *those of V<V_1_. When the conformer volume increases from V<V_1 _to V = V_1_, the "large unique shapes" for V = V_1 _explain newly added shape space whereas the "small unique shapes" for V = V_1 _represent the shape space not present for that volume. The unchanged portion of the shape space is explained by the "shared unique shapes" for V = V_1_. Figure 5 schematically illustrates the shape space expansion upon a successive increase in the conformer volume. Note that smaller *ST^thresh ^*values were used for clustering as the volume increases (as represented by larger circles) to maintain the number of unique shapes to a manageable size and to reflect the *ST^thresh ^*value used in Figure 3 for V_1_.

**Figure 6:**
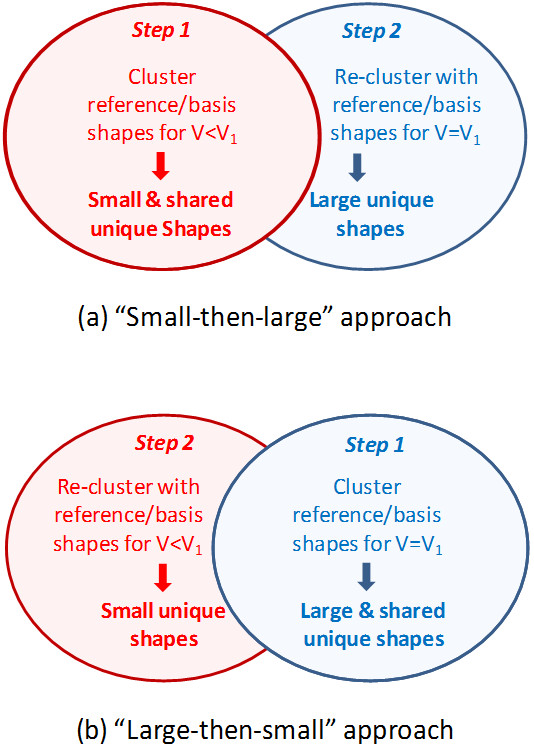
**Two different approaches used to generate the unique shapes between V = V_1 _and V<V_1_, depending on which shape space is clustered first**.

The "unique shapes" for each volume were computed using two different clustering strategies, the "small-then-large" approach and the "large-then-small" approach, as depicted in Figure 6, and detailed procedures are described in the *Materials and Methods *section. In the "small-then-large" approach [Figure 6(a)], the shape space of the conformers of V<V_1 _was first explored at the *ST^thresh ^*value for V_1 _to look for newly added shape space when the conformer volume increases to V_1_. That is, the small and shared unique shapes for V = V_1_, which cover the shape space spanned by conformers of V<V_1_, were first generated by clustering all reference and basis shapes for V<V_1_, and then the identified unique shapes were re-clustered with the reference and basis shapes for V = V_1 _to find the large unique shapes. On the contrary, in the "large-then-small" approach [Figure 6(b)], the large and shared unique shapes for V = V_1 _were determined first, by using the previously determined reference shapes for V = V_1_, and then the reference and basis shapes for V<V_1 _were used to re-cluster to identify the small unique shapes.

**Figure 7:**
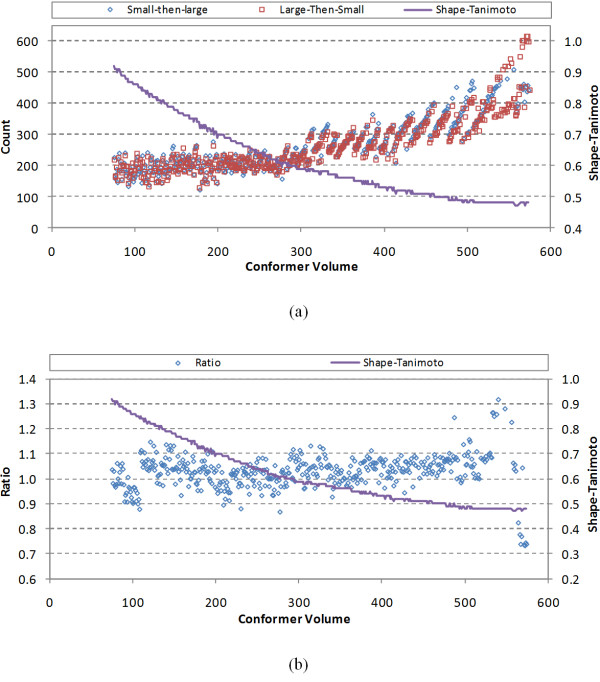
**Unique shape counts**. (a) The number of unique shapes generated by the "small-then-large" method and the "large-then-small" method, and (b) the ratio of "small-then-large" to "large-then-small" unique shapes as a function of conformer volume.

The two methods resulted in two different sets of the unique shapes for each volume. The unique shape counts for both sets and the ratio between them are plotted in Figure 7(a), as a function of the conformer volume. Because both methods deal with the identical shape space, they are expected to give a number of unique shapes similar to each other; however, since reference shapes were selected randomly without any attempt to optimally minimize or maximize their count, these counts cannot be expected to be the same. As shown in Figure 7, the unique shape counts for the two sets tended to differ by 0-10%, although their ratio varied from 0.7 to 1.3 (especially for V>500, where the conformer populations were not as numerous). This tendency may be explained by the fact that lesser volumes consider reference and basis shapes that may be considerably closer together due to larger *ST^thresh ^*values. This suggests that using the larger volume reference shapes first resulted in a more efficient shape space description (*i.e*., fewer reference shapes), when considering the union of the collective shape space for the volume range. Nonetheless, Figure 7 shows, as expected, that the total number of unique shapes gradually increases as a function of the conformer volume and its *ST^thresh ^*value, indicating an overall expansion of shape space across the volume range irrespective of the change in ST value used (*i.e*., shape space is growing faster than the decrease in ST value as a function of volume to achieve a maximum of 200 reference shapes).

**Figure 8:**
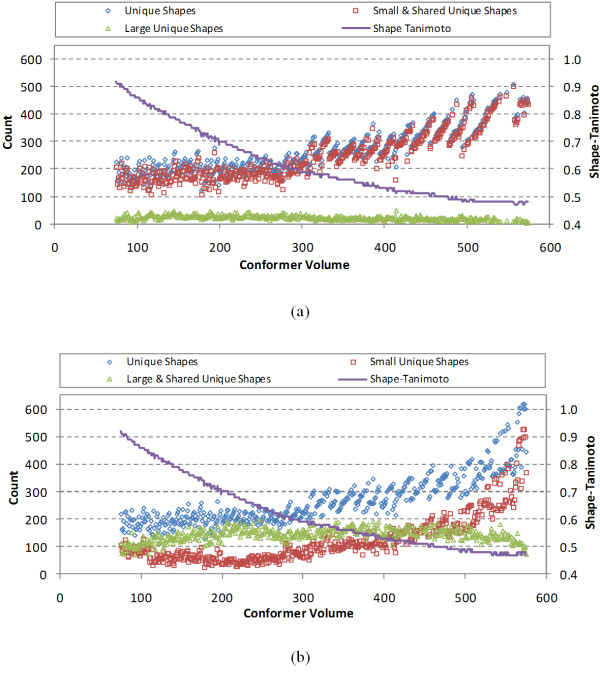
**The number of unique shapes, small unique shapes, and large unique shapes generated using (a) the small-then-large method and (b) the large-then-small method**.

**Figure 9:**
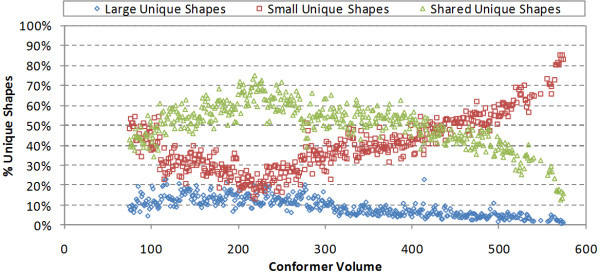
**The percentages of the large unique shapes, small unique shapes, and shared unique shapes, being the percentage of space not covered by either large or small unique shapes [*i.e*., shared = 1.0 - (large + small)], as a function of the conformer volume**.

Figure 8 displays the number of large unique shapes, small unique shapes, and shared unique shapes for each volume, while Figure 9 shows their proportions of the total unique shapes, which were estimated using the following equations:(3)(4)(5)

Note that the value of *ST^thresh ^*affects the counts of reference, basis, and unique shapes, because it determines the distance between clusters. However, the percentages of these counts plotted in Figure 9 are essentially equivalent to the fractions of the shape space that the individual counts represent, and hence, they may be considered to be independent of the *ST^thresh ^*value.

There are a number of interesting observations one can make from these graphs. In Figure 7 and Figure 8 there is a banded behavior, indicated previously in Figure 3, which looks like a series of lines spaced further apart as the volume increases. This is due to the steady growth in shape space as volume increases and the use of 0.01 decrements of *ST^thresh^*. Whenever the *ST^thresh ^*decreases by 0.01, a corresponding significant decrease in counts occurs. When the *ST^thresh ^*value changes less, or does not change at all, the lines appear to be wider apart, reflecting just the growth in shape space due to volume.

Another interesting observation in Figure 8(a), one can see that the absolute count of large unique shapes stays relatively constant in the volume range, with an average count and standard deviation of 22.2 +/- 7.8 and a mode of 24. There is a shallow maximum at volume 145 Å^3 ^followed by a relatively slow overall decline over the rest of the volume range. This decline appears most evident when the volume is beyond volume 305 Å^3^, perhaps due to the truncation of shape space considered as represented by the rapid reduction in conformer count at larger volumes and the fact that a maximum of non-hydrogen atom count occurs at 26.

Similar to the large unique shapes in Figure 8(a), the large and shared unique shapes in Figure 8(b) show a similar banded behaviour across most of the volume range, with a reference count mean and standard deviation of 144.4 +/- 23.7 and a mode of 140. There is a barely evident maximum volume at volume 228 Å^3 ^and a slightly noticeable dip at volume 261 Å^3^, prior to resuming the similar narrow band of large and shared unique shapes. This may suggest that the growth of large and shared shape space is relatively constant as a function of PubChem contents.

The small and shared unique shapes completely dominate in Figure 8(a), being nearly the same as the total count of unique shapes across the entire volume; however, the small unique shapes in Figure 8(b) show a very shallow minimum at about volume 200 Å^3 ^prior to significantly increasing as a function of volume. This may suggest that the overall size of PubChem shape space slows (as a function of the rate of changing ST) after a point, with large unique shapes contributing less and less to the overall shape diversity across the full volume range as the total shape space that can be represented by larger shapes diminishes. One can see this to some extent in Figure 9, where the percentage of shared shape space is "Λ"-shaped, reaching a maximum of 73% at volume 217 Å^3 ^and then steadily diminishes as a function of volume as the percentage of shape space of smaller shapes dominates. Again, it is reasonable to suggest that this observation is an artifact of the PubChem contents and not representative of what one might find if significantly more larger chemical structures were considered in the range of 30-50 non-hydrogen atoms. (*i.e*., if the non-hydrogen atom count maximum was not at 26, but continued to grow until the maximum considered of 50.)

**Figure 10:**
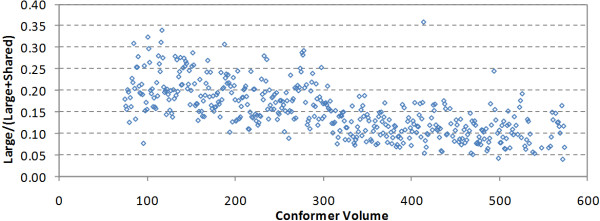
**The ratio of the percent large unique shapes to the sum of the the percent large and shared unique shapes**.

To further demonstrate this, Figure 10 shows the ratio of the fraction of the large unique shapes to the sum of the fractions of the large and shared unique shapes, which is a measure of how much of the shape space spanned by the conformers of a particular volume is not shared by the conformers smaller than that volume. For 75 Å^3 ^≤ V ≤ 100 Å^3^, the mean value of the ratio was 0.19, indicating that ~20% of the shape space spanned by the conformers of a particular volume in this range is unique to that particular volume, and that the other 80% is shared by the conformers smaller than that volume. The ratio decreases with the conformer volume, and the mean value for 550 Å^3 ^≤ V ≤ 575 Å^3 ^was 0.11, indicating the rate of the shape space growth decreases as the conformer volume increases, relative to the PubChem chemical structure contents.

## Conclusion

The shape diversity of the biologically relevant conformer space of molecules and conformers was investigated using 16.4 million molecules in the PubChem Compound database (as of January 2008), covering non-hydrogen atom counts up to 50 and effective rotors up to 15, as represented by 1.46 billion diverse conformers. After binning the conformers according to their volume, cluster analysis was performed to get a maximum count of non-redundant reference shapes, representing the shape space spanned by the conformers for a particular unit volume. The *ST^thresh ^*value, which defines the maximum shape similarity between any two reference shapes for that volume, gradually decreased as the conformer volume increased. There was no apparent correlation between the count of conformers clustered and the shape diversity found. Furthermore, an analysis was performed to examine the rate of increase of new reference shapes as a function of volume and the percentage of shape space unique to a particular volume. Generally speaking, the rate of addition of new reference shapes as a function of increasing volume was relatively constant across the range of volumes considered; however, the ability of a particular volume to explain the shape diversity spanned by lesser volumes increased up to a point and then decreased, ranging between 40-70% of all unique shapes for most of the considered volume range (Figure 9).

Some of the results from this analysis should be considered an artifact of the contents of PubChem in that the population as a function of molecular size peaks at 26 non-hydrogen atoms and then rapidly declines. An exhaustive analysis of all "reasonable" theoretically possible molecules resulting from larger molecules may provide a different trend. As such, the results of this analysis should be considered a conservative estimate.

While it is unfortunate that the PubChem shape space is truncated based on what is possible (due to the diminishing count of chemical structures with non-hydrogen atom counts greater than 26), one does see substantial evidence that shape space grows uniformly with a smoothly decreasing *ST^thresh ^*and increasing molecular volume. One also sees that keeping the count of reference shapes at a maximum for a given volume as an approach and analysis can allow one to achieve an understanding as to how diverse shape space is as a function of shape similarity. The apparent lack of dependence of the reference shape count with respect to the count of conformers represented by a given volume demonstrates how redundant shape space is across the volume range; however, we believe that the *ST^thresh ^*curve in Figure 3 may actually be linear or approach it, if provided an exhaustive set of theoretically possible but reasonable chemical structures, as the chemical structure shape possibilities surely are more diverse than the limited population of chemical structures available in PubChem for non-hydrogen atom counts greater than thirty.

## Materials and methods

### 1. Biologically relevant molecules in the PubChem Compound database

From the PubChem Compound database [15-18], a subset of biologically relevant molecules that satisfy the following restrictions was downloaded for subsequent conformer generation:

(1) Molecules only with a single covalent component were considered, since each component of mixtures and complexes has a unique Compound ID.

(2) Salts were not included because their parent molecules are also in the PubChem database.

(3) Molecules with a non-organic element were not included because they are not compliant with the 94 s variant of the Merck molecular force-field (MMFF94s), which was used for conformer generation (without coulomb interaction terms). For the same reason, molecules with an MMFF94 s unparameterized element type (*e.g*., hyper-valent species) were removed.

(4) Molecules that are too big or too flexible cannot have their conformational space properly sampled. Therefore, the non-hydrogen atom count was limited to a maximum of 50 and the effective rotor count was limited to 15. The effective rotor count, given by the following equation, takes into account the additional flexibility due to non-aromatic rings in a molecule,(6)

where *nr_effective _*is the number of effective rotors, *nr *is the number of rotatable bonds, and *nnara *is the number of "non-aromatic" *sp^3^*-hybridized ring atoms.

(5) Molecules with more than 6 undefined stereocenters were also removed because they need substantial computational resources to consider.

### 2. Conformer generation

The OMEGA C++ application programming interface (API) [19] was used to generate conformers for the molecules in PubChem and the Shape C++ API [12] was used to compute conformer analytic volumes. In a recent study [20], a set of optimal values for some important OMEGA parameters was determined for PubChem 3-D conformer generation. This parameter set was employed for conformer generation in the present study. The MMFF94 s force-field without the coulomb interaction terms were used with the energy window limited to 25 kcal/mol. The number of conformers generated in the torsion search step was limited to 100,000 conformers. When undefined stereocenters were present, each stereo isomer was independently considered (maximum of 100,000 conformers each for up to 32 different SP3/SP2 stereo isomers) and all produced conformers combined. Conformers were then clustered using the root-mean-square distance [rounded to the nearest 0.2 increment (from 0.4 to 2.4)] given by the following equation [20]:(7)

where *nr_effective _*is the number of effective rotors and *n_nha _*is the number of non-hydrogen atoms in a molecule. The maximum number of conformers in a conformer model for each molecule was limited to 500. If clustering resulted in more than 500 conformers, the clustering RMSD was incremented by 0.2 and the conformers re-clustered, repeating until 500 or fewer conformers were achieved. Post processing of the conformer models was performed. This included full energy minimization of all hydrogen atom locations (all non-hydrogen atoms were kept frozen). Subsequent analysis removed any conformers with atom-atom "bumps", being cases where the steric van der Waals interaction energy was greater than 25 kcal/mol.

### 3. General descriptions of the partition-clustering algorithm

Due to the rather large number of conformers involved, a "divide and conquer" approach with a multistage partition-based clustering algorithm (as shown in Figure 2) was employed. In the first phase of the partition-clustering algorithm, conformers were split into manageable sets (or partitions), each containing a certain number of conformers (*N*^*setsize *^= 50,000). Conformers in each set were randomly sampled such that no two selected conformers had a ST distance closer than the shape diversity threshold (*ST^thresh^*). The selected conformers were retained for future analysis, as cluster representatives, while the others were considered redundant and discarded. If the count of selected conformers in a given partition was greater than , the partition-clustering procedure with these conformers was repeated at a decreased *ST^thresh ^*value. After all conformer sets were sampled using *ST^thresh^*, all the conformers from each set were then combined and re-sampled as described above (*e.g*., divided up into partitions and sampled). When the total number of clusters became smaller than , a "non-partition" clustering was performed to eliminate the redundancy among cluster representatives from the different partitions. If the number of clusters from the non-partition clustering procedure was greater than , the clustering was repeated at a decreased *ST^thresh ^*value. In the end, the ST scores between any two conformers in the final reference set cannot be closer than the *ST^thresh ^*value. A final step involved comparing the reference set with all conformers represented by the reference set.

This procedure achieves several things. Firstly, it breaks up many millions of conformers into manageable sets. Secondly, it allows the shape diversity threshold to be dynamically decreased for individual conformer sets. Thirdly, it reduces a very large number of conformers to a manageable set of conformers that represent all possible shapes present.

#### 3.1. Partition-clustering of conformers of a given volume

To study the shape diversity for a given volume, the conformers of the same volumes were partition-clustered, based on the procedures outlined in the previous section.

(1) The 1.46 billion conformers were grouped according to their volumes rounded to the nearest integers.

(2) The conformers for a given volume were partition-clustered until the total number of clusters became less than  = 6,000. The set of the seed conformers representing these clusters were considered the "basis shapes" for that volume.

(3) The non-partition clustering was performed with the basis shapes, decreasing *ST^thresh ^*value 0.01 at a time, until the number of cluster representatives became less than  = 200.

#### 3.2. Generation of unique shapes

To investigate the shape space redundancy between different volumes, the unique shapes (Figures 4 and 5) for each volume were generated using two different clustering schemes: (1) the "small-then-large" method and (2) the "large-then-small" method (Figure 6). In the small-then-large method, the unique shapes for V = V_1 _were generated from clustering of the reference and basis shapes for V<V_1_, and re-clustering with the reference and basis shapes for V = V_1_, to locate those shapes unique only to the current volume. On the contrary, in the large-then-small method, the unique shapes were generated by pooling the reference shapes for V = V_1_, and re-clustering with the reference and basis shapes for V<V_1_, to locate only those shapes that are unique to lesser volumes.

##### 1. The "small-then-large" approach

(1) Pool all reference shapes of V<V_1 _and partition-cluster them at .

(2) Cluster the partition-clustered reference shapes [from step (1)] at .

(3) Pool all basis shapes for V<V_1 _and partition-cluster them at .

(4) Fill cluster holes in the clustered reference shapes [from step (2)], by re-clustering them with the partition-clustered basis shapes [from step (3)] at .

(5) Fill cluster holes in the clusters from step (4) with the reference shapes for V = V_1_.

(6) Fill cluster holes in the clusters from step (5) with the basis shapes for V = V_1_.

##### 2. The large-then-small approach

(1) Pool all reference shapes of V = V_1_.

(2) Pool all reference shapes of V<V_1 _and partition-cluster them at .

(3) Cluster the partition-clustered reference shapes [from step (2)] at .

(4) Pool all basis shapes for V<V_1 _and partition-cluster them at .

(5) Fill cluster holes in the clustered reference shapes [from step (3)], by re-clustering them with the partition-clustered basis shapes [from step (4)] at .

(6) Fill cluster holes in the clustered reference shapes for V = V_1 _[from step (1)] with the clusters from step (5).

## Competing interests

The authors declare that they have no competing interests.

## Authors' contributions

EEB performed most of the research. SK wrote the first draft and introduced the concept of small and large unique shapes to analyze the data. SHB reviewed the final manuscript. All authors read and approved the final manuscript.
